# A comprehensive characterization of myocardial and vascular phenotype in pediatric chronic kidney disease using cardiovascular magnetic resonance imaging

**DOI:** 10.1186/s12968-018-0444-0

**Published:** 2018-03-29

**Authors:** Mun Hong Cheang, Nathaniel J. Barber, Abbas Khushnood, Jakob A. Hauser, Gregorz T. Kowalik, Jennifer A. Steeden, Michael A. Quail, Kjell Tullus, Daljit Hothi, Vivek Muthurangu

**Affiliations:** 1 Centre for Cardiovascular Imaging, UCL Institute of Cardiovascular Science, 30 Guilford Street, London, WC1N 1EH UK; 2 Great Ormond Street Hospital, London, UK

**Keywords:** Chronic renal failure, Pediatrics, Arterial stiffness, Systemic vascular resistance, Hypertension, Myocardial impairment

## Abstract

**Background:**

Children with chronic kidney disease (CKD) have increased cardiovascular mortality. Identifying high-risk children who may benefit from further therapeutic intervention is difficult as cardiovascular abnormalities are subtle. Although transthoracic echocardiography may be used to detect sub-clinical abnormalities, it has well-known problems with reproducibility that limit its ability to accurately detect these changes. Cardiovascular magnetic resonance (CMR) is the reference standard method for assessing blood flow, cardiac structure and function. Furthermore, recent innovations enable the assessment of radial and longitudinal myocardial velocity, such that detection of sub-clinical changes is now possible. Thus, CMR may be ideal for cardiovascular assessment in pediatric CKD. This study aims to comprehensively assess cardiovascular function in pediatric CKD using CMR and determine its relationship with CKD severity.

**Methods:**

A total of 120 children (40 mild, 40 moderate, 20 severe pre-dialysis CKD subjects and 20 healthy controls) underwent CMR with non-invasive blood pressure (BP) measurements. Cardiovascular parameters measured included systemic vascular resistance (SVR), total arterial compliance (TAC), left ventricular (LV) structure, ejection fraction (EF), cardiac timings, radial and longitudinal systolic and diastolic myocardial velocities. Between group comparisons and regression modelling were used to identify abnormalities in CKD and determine the effects of renal severity on myocardial function.

**Results:**

The elevation in mean BP in CKD was accompanied by significantly increased afterload (SVR), without evidence of arterial stiffness (TAC) or increased fluid overload. Left ventricular volumes and global function were not abnormal in CKD. However, there was evidence of LV remodelling, prolongation of isovolumic relaxation time and reduced systolic and diastolic myocardial velocities.

**Conclusion:**

Abnormal cardiovascular function is evident in pre-dialysis pediatric CKD. Novel CMR biomarkers may be useful for the detection of subtle abnormalities in this population. Further studies are needed to determine to prognostic value of these biomarkers.

**Electronic supplementary material:**

The online version of this article (10.1186/s12968-018-0444-0) contains supplementary material, which is available to authorized users.

## Background

Cardiovascular events are a leading cause of death in pediatric chronic kidney disease [1] (CKD). Two-dimensional transthoracic echocardiography (TTE) is conventionally used to assess the heart in children with CKD. Several TTE markers have been used as indicators of increased risk including: left ventricular (LV) hypertrophy (LVH), LV dysfunction and abnormal myocardial strain [2–4]. However, TTE can be hampered by inaccurate measurement of volumes and mass [5], as well as operator dependence.

Cardiovascular magnetic resonance (CMR) cine imaging is the reference standard method of assessing LV volumes, ejection fraction (EF) and mass [6]. Novel CMR including tissue phase mapping (TPM) to assess myocardial velocities [7] and high temporal resolution phase contrast CMR (PCMR) to measure mitral inflow velocities and cardiac timing intervals are also now available. Furthermore, CMR can be used to characterize the vasculature by combining flow measurements with simultaneously acquired blood pressure (BP) [8]. Thus, CMR offers a comprehensive method of assessing myocardial and vascular phenotype in pediatric CKD.

The objectives of this study were to i) evaluate any differences in vascular and cardiac phenotype between healthy children and those with CKD, ii) determine the relationship between vascular and cardiac phenotype and any additional effect of CKD, iii) assess the relationship between markers of renal disease and cardiovascular phenotype.

## Methods

### Study population

The study population consisted of 120 children: 100 with confirmed CKD (stages 1–5) and 20 healthy volunteers. The CKD patients were divided into 3 groups based on CKD stage: Mild (CKD stage 1–2), Moderate (CKD stage 3) and Severe (CKD stage 4–5). The study was powered to detect differences in mean arterial BP (MBP) between the groups (including healthy controls). For between group comparisons, a sample size of 14 children per group provided 90% power (*p* < 0.05) to detect a difference of 3 mmHg in MBP (assuming a normal MBP of 82 mmHg, and a variance of 40 mmHg [9]). The group size was increased to 20 for redundancy. In addition, we increased the population size of the mild and moderate CKD groups to 40 children in order to improve our spread of estimated glomerular filtration rate (eGFR) data points for correlation analysis.

All patients were recruited from the CKD and hypertension clinics at Great Ormond Street Hospital, London. Healthy subjects were recruited by advertising within the hospital and a detailed history was taken from the parents to ensure there was no significant medical history. Exclusion criteria were: i) age < 7 or > 18 years, ii) congenital structural heart disease or primary myocardial disease, iii) primary renovascular disease, iv) active vasculitis v) cardiac arrhythmia, vi) medical devices precluding CMR, vii) history of current or previous renal replacement therapy and viii) acutely deteriorating renal function. This study was approved by UK national research ethics service (National Research Ethics Service Committee London, London Bridge, reference: 15/LO/0213). Informed parental consent and patient assent was obtained from all participants.

All CKD participants had blood and urine tests as part of their outpatient care including: haemoglobin (Hb), urea, creatinine, electrolytes, calcium, phosphate, intact parathyroid hormone level (PTH), and urine albumin to creatinine ratio measurements. The eGFR was estimated by modified Schwartz formula. Neither blood nor urine tests were performed for healthy controls.

### Imaging protocol

Imaging was performed on a 1.5 T CMR system (Avanto; Siemens Healthineers, Erlangen, Germany) with vector cardiographic gating. All CMR images were processed using in-house plug-ins developed for open-source DICOM software OsiriX [10] (Osirix Foundation, Geneva, Switzerland). All images were analyzed by the same individual, who is a CMR trained cardiologist (MC) with 5 years of CMR experience. No gadolinium- contrast was given.

### Left ventricular volumes and mass

LV volumes were assessed using short axis multi-slice free-breathing real-time radial k-t SENSE balanced steady state free precision sequence sequence [11]. Scan parameters - FOV: ≈350 mm, matrix: 128 × 128, voxel size: ≈2.7 × 2.7 × 8 mm, TE/TR: ≈1.1/ ≈2.2 msec, Flip angle: 40°, acceleration factor: 8, temporal resolution: ≈36 msec. LV endocardial borders were manually traced at end-diastole and end-systole to evaluate end-diastolic volume (EDV) and end-systolic volume (ESV). Stroke volume (SV) was obtained by subtracting ESV from EDV. All ventricular volumes were indexed to body surface area (BSA). LV EF was assessed SV/LVEDV) × 100.

Epicardial LV borders were manually traced in end-diastole and combined with endocardial borders to obtain LV mass (LVM). The effect of body size on LV mass was controlled by both indexing to height to the power of 2.7 (LVMht^2.7^) [12] and dividing LVM by EDV to calculate mass volume ratio (MVR) [13].

The right atrial and left atrial areas were measured in diastole from 4-chamber view (using the same sequence) and indexed to BSA.

### Cardiac timing and inflow velocities

LV outflow tract and mitral inflow velocities were assessed with a free-breathing high temporal resolution real-time UNFOLD-SENSE spiral PCMR sequence. This sequence has previously been validated against Doppler echocardiography [14]. Scan parameters - FOV: ≈450 mm, matrix: 128 × 128, voxel size: ≈3.5 × 3.5 × 7 mm, TE/TR: ≈1.97/ ≈7.41 msec, flip angle: 20°, VENC: 150 m/s, acceleration factor: 10, temporal resolution: ≈15 ms. The imaging plane was positioned so that both the mitral inflow and LV outflow tract were imaged in the short axis.

The mitral valve orifice and LV outflow tract were manually segmented from the resultant inflow and outflow curves to obtain the isovolumic relaxation time (IRT), isovolumic contraction time (ICT) and ejection time (ET) as previously described [14]. Myocardial performance index (Tei) was calculated as the sum of ICT and IRT divided by ET. Peak early (E) and atrial (A) diastolic velocities were measured from the inflow curves.

### Myocardial velocities

LV myocardial velocities was measured using a free breathing self-navigated golden-angle spiral TPM sequence planned in the mid LV short axis view [7, 15]. Scan parameters - FOV: ≈400 mm, matrix: 192 × 192, voxel size: ≈2.1 × 2.1 × 8 mm, TE/TR: ≈3.51/ ≈11.7 msec, flip angle: 15°, respiratory navigation efficiency: 30%, scan time: ≈7-8 min, temporal resolution: ≈23 msec.

All TPM indices were measured from a mid LV short axis slice. Each acquisition produces a magnitude image and 3 phase images (in the x-, y- and z- direction). The epi- and endocardial borders were manually segmented on the magnitude image for all frames and regions of interest were transferred to the accompanying phase images. From these masked phase images, radial velocity was calculated by transforming the x and y-direction velocities to an internal polar coordinate system using the LV center of mass as a reference point [7, 15]. The longitudinal velocity was taken as the z-direction velocity. Global radial and longitudinal LV velocities were calculated by averaging velocities across the segmented LV slice for a given direction. The magnitude of the peak systolic (S′), early diastolic (E’) and late diastolic (A’) velocities were measured from the velocity-time curves. E’/A’ ratio was the ratio of longitudinal E’ over longitudinal A’ velocity. The longitudinal E/E’ ratio was obtained by dividing the mitral E wave velocity by longitudinal peak early diastolic velocity.

### Aortic flow

Aortic flow assessment was performed using a breath held retrospectively-gated, spiral SENSE PCMR just above the sinotubular junction [16]. Scan parameters - FOV: ≈400 mm, matrix: 256 × 256, voxel size: ≈1.6 × 1.6 × 5 mm, TE/TR: ≈2.1/≈8.0 msec, flip angle: 25°, acceleration factor: 3, breath hold time: 4-8 s, temporal resolution: ≈32.0 msec.

The aorta was segmented using a semi-automatic vessel edge detection algorithm with manual operator correction. The SV was derived from the flow curve and multiplied by heart rate to calculate cardiac output (CO). The maximum (AoMax) and minimum (AoMin) cross-sectional areas of the ascending aorta over the cardiac cycle were also recorded.

### Blood pressure measurement

Brachial systolic, diastolic and mean arterial BPs (SBP, DBP, MBP) were measured using a CMR compatible oscillometric sphygmomanometer (Datex Ohmeda; General Electric Healthcare, Boston, Massachusetts, USA) during CMR image acquisition. This enabled optimum combination of BP and flow data for calculation of vascular indices. All BP measurements were acquired with an appropriate sized arm cuff after the subject had been lying in the scanner for at least 10 min. Pulse pressure (PP) was the difference between SBP and DBP.

### Measures of vascular characteristics

Total systemic vascular resistance (SVR) was calculated by dividing MBP by cardiac output (CO) [8]. Total arterial compliance (TAC) was calculated using a two-element windkessel model described previously [8]. Briefly, aortic flow curves were inputted into the model with measured SVR. The compliance was tuned so that pulse pressure generated by the model equaled measured pulse pressure. Ascending aortic strain (AoS) was calculated as (AoMax – AoMin)/AoMin (expressed as a percentage). Local arterial stiffness was assessed by calculating ascending aortic compliance (AoC) = AoS/pulse pressure [17].

### Statistics

Statistical analyses were performed using Stata 13 (StataCorp, College Station, Texas, USA). Data were examined for normality using Shapiro-Wilk test and non-normally distributed data was transformed using a zero-skewness log transform prior to analysis. Descriptive statistics were expressed as mean (± standard deviation) or geometric mean (± geometric standard deviation) if data was log transformed. Between CKD group differences in vascular and cardiac phenotype were assessed using analysis of variance (ANOVA) (objective 1). Levene’s test was used to assess for homogeneity of variances across the groups and Welch’s correction was applied for non-homogeneous variance. Six groups of statistical tests comparing healthy controls to patients were performed in the following domains - baseline characteristics, blood pressure, vascular phenotype, global cardiac structure and function, cardiac timings and mitral velocities, and tissue phase mapping. Each group of tests was considered a single family of statistical inferences and the family-wise error rate was controlled using Bonferroni correction. The corresponding corrected critical *p*-values are listed in the respective tables. Post-hoc pairwise comparison was performed on parameters significantly different on ANOVA testing. The Bonferroni method was used to adjust for potential Type 1 errors in the pairwise comparisons. Spearman’s correlation coefficient (rho) was used to determine the relationship between specific vascular and cardiac phenotypic markers (objective 2) that were significant on ANOVA testing. Multi-variable ANOVA models were also constructed to examine the independent effect of CKD group. Spearman’s correlation coefficient was also used to determine the relationship between markers of renal disease severity - eGFR, hemoglobin (Hb) and PTH and significant abnormal cardiac and vascular indices - MVR, IRT, S′, E’, MBP, SVR (objective 3). Multi-variable linear regression was used to determine the independent effect of renal severity on cardiac markers by controlling for vascular phenotype. Only patients with recent blood tests were included into models involving blood indices. A *p* value < 0.05 was considered statistically significant, except where Bonferroni correction was applied as indicated. Indices not statistically different to healthy subjects have been described as ‘normal’.

## Results

### Demographics

The CKD cohort (*n* = 100) consisted of 40 children with mild CKD (stage 1–2: eGFR 78.6 ± 8.8 ml/min/1.73 m^2^), 40 children with moderate CKD (stage 3: eGFR 48.3 ± 8.4 ml/min/1.73 m^2^) and 20 children with severe CKD (eGFR 19.2 ± 6.5 ml/min/1.73 m^2^). Twenty-five (25%) children (mild CKD: 10, moderate CKD: 9; severe CKD: 6) had a prior diagnosis of hypertension and were on treatment (Table 1). The two most common causes of CKD were congenital abnormalities of the kidney and urinary tract (*n* = 60, 60%) and renal dysplasia (*n* = 11, 11%). All other causes represented < 10% of the population. Eight CKD children had undergone a previous nephrectomy. No children had previously received or were currently receiving renal replacement therapy as per exclusion criteria. None of the healthy subjects (*n* = 20) had any significant past medical history nor were they taking any medications. There were no differences in age, BSA or sex distribution between groups (Table 1). As expected, markers of renal function (urea, creatinine, eGFR and urine albumin/creatinine ratio) were worse in the latter CKD stages, with the most abnormal results observed in the severe CKD group (*p* < 0.03). The severe CKD group also had highest parathyroid hormone (PTH) (*p* = 0.01). There was no difference in Hb between the groups (*p* = 0.57). Outline of blood and urine tests results of study population can be seen in Additional file 1: Table S1.

**Table 1:** Demographics and Baseline Characteristics of study population

	Healthy Controls (*n* = 20)	Mild CKD (*n* = 40)	Moderate CKD (*n* = 40)	Severe CKD (*n* = 20)	*P*-value
Age (years)^a^	12 ± 1.3	11 ± 1.3	12 ± 1.3	13 ± 1.3	0.12
Sex (% male)	40%	38%	23%	45%	0.42
Height (cm)	162 ± 18	150 ± 17	152 ± 18	151 ± 18	0.075
Weight (kg)^a^	53 ± 1.4	43 ± 1.5	45 ± 1.5	44 ± 1.4	0.22
Body Mass Index (kg/m^2^)^a^	20 ± 1.1	19 ± 1.2	20 ± 1.2	20 ± 1.1	0.77
Body Surface Area (m^2^)	1.6 ± 0.32	1.4 ± 0 .33	1.4 ± 0.35	1.4 ± 0.3	0.16
Hemoglobin (g/L)		132 ± 13	133 ± 16	128 ± 11	0.57
Urea (mmol/L)		5.4 ± 1.2	8.6 ± 2.5	18.0 ± 5.2	< 0.001^†^
Creatinine (umol/L)^a^		68 ± 1.2	117 ± 1.2	305 ± 1.4	< 0.001^†^
eGFR (ml/min/1.73 m^2^)		79 ± 8.8	48 ± 8.4	19 ± 6.5	< 0.001
Calcium-Phosphate product (mmol^2^/L^2^)		3.4 ± 0.45	3.4 ± 0.39	3.5 ± 0.56	0.40
Intact parathyroid hormone (pmol/L)^a^		3.5 ± 3.2	3.7 ± 2.4	8.5 ± 2.4	0.011
Urine Albumin/ Creatinine Ratio (mg/mmol)^a^		4.7 ± 3.7	22 ± 6.2	261 ± 2.5	< 0.001
Angiotensin converting enzyme inhibitor		28%	40%	15%	0.13
Calcium Channel Blocker		5%	13%	15%	0.38
Beta-Blocker		5%	5%	20%	0.09
Aldosterone antagonist (Spironolactone)		3%	0%	0%	0.47

Bonferroni correction applied- *p* < 0.003 is considered significant

*Abbreviations*: *CKD* chronic kidney disease, *eGFR* estimated globular filtration rate

^a^Logarithmic transformation was applied

^†^ANOVA Welch (W) test was used. 10% of CKD patients were on 2 or more anti-hypertensive agents

### Vascular characteristics

The DBP, DBP centile and MBP were significantly different between the groups (*p* < 0.001, Table 2) and were elevated in all CKD groups compared to controls (*p* < 0.05) on pairwise comparison. However, there were no significant differences between the CKD groups. There were also no group differences in SBP and PP (*p* ≥ 0.13), although there was a trend for SBP centile (*p* = 0.08).

**Table 2:** Blood pressure profile of study population

	Healthy Controls (*n* = 20)	Mild CKD (*n* = 40)	Moderate CKD (*n* = 40)	Severe CKD (*n* = 20)	*P*-value
Systolic BP (mmhg)^a^	109 ± 1.1	113 ± 1.1	116 ± 1.1	116 ± 1.1	0.14
Systolic BP percentile^a^	33 ± 2.5	58 ± 1.7	61 ± 1.8	64 ± 1.7	0.08^†^
Diastolic BP (mmhg)^a^	53 ± 1.2	63 ± 1.2	65 ± 1.2	69 ± 1.2	< 0.001
Diastolic BP percentile^a^	17 ± 2.3	45 ± 1.9	48 ± 1.8	60 ± 1.6	< 0.001^†^
Mean BP (mmhg)	78 ± 7.8	84 ± 8.8	87 ± 9.9	89 ± 8.8	< 0.001
Pulse Pressure (mmhg)	55 ± 13	50 ± 10	51 ± 9.5	48 ± 12	0.13
Heart Rate (BPM)^a^	72 ± 1.2	75 ± 1.2	72 ± 1.2	74 ± 1.2	0.75

Bonferroni correction applied- *p* < 0.007 is considered significant

*Abbreviations*: *CKD* chronic kidney disease, *BP* blood pressure, *BPM* beats per minute

^a^Logarithmic transformation was applied

^†^ANOVA Welch (W) test was used

The SVR was also significantly different between the groups (*p* = 0.004, Table 3). On pairwise comparison, both moderate and severe CKD groups were elevated compared to controls (*p* ≤ 0.014). However, there were no significant differences in SVR between the CKD groups. Importantly, there were no group differences in TAC, local measures of ascending aorta stiffness (AoS and AoC) or cardiac output (Table 3).

**Table 3:** Vascular phenotype in CKD

	Healthy Controls (*n* = 20)	Mild CKD (*n* = 40)	Moderate CKD (*n* = 40)	Severe CKD (*n* = 20)	*P*-value
SVR (WU.m^2^)^a^	21 ± 1.3	24 ± 1.2	26 ± 1.2	26 ± 1.2	0.004
TAC (ml/mmHg. m^2^.10^2^)^a^	52 ± 1.3	55 ± 1.2	54 ± 1.3	58 ± 1.3	0.56
AoS (%)^a^	46 ± 1.5	52 ± 1.6	53 ± 1.6	51 ± 1.5	0.74
AoC (%mmHg^−1^.10^2^)^a^	86 ± 1.6	108 ± 1.6	105 ± 1.7	111 ± 1.7	0.33
CO^a^ (l/min/m^2^)	3.6 ± 1.2	3.5 ± 1.2	3.4 ± 1.2	3.4 ± 1.2	0.61

Bonferroni correction applied- *p* < 0.01 is considered significant

*Abbreviations*: *CKD* chronic kidney disease, *SVR* systemic vascular resistance, *TAC* total arterial compliance. *AoS* ascending aortic strain, *AoC* ascending aortic compliance, *CO* cardiac output

^a^Logarithmic transformation was applied

### Cardiac geometry and global function

There was a significant difference in MVR (a marker of LV remodeling) between the groups (*p* = 0.003, Table 4). On pairwise comparison, only the severe CKD group was significantly different from healthy subjects and all other CKD groups (*p* ≤ 0.034).

**Table 4:** Cardiac structure and global function in CKD

	Healthy Controls (*n* = 20)	Mild CKD (*n* = 40)	Moderate CKD (*n* = 40)	Severe CKD (*n* = 20)	*P*-value
LVEDV (ml/m^2^)	74 ± 11	68 ± 9.1	68 ± 10	65 ± 9.7	0.03
LVESV (ml/m^2^)^a^	23 ± 1.3	21 ± 1.3	20 ± 1.3	19 ± 1.3	0.13
LVSV (ml/m^2^)^a^	50 ± 1.1	46 ± 1.1	47 ± 1.2	46 ± 1.2	0.18
EF (%)	68 ± 4.6	68 ± 6.1	69 ± 6.7	70 ± 5.8	0.72
LVMht^2.7^ (g/m^2.7^)^a^	21 ± 1.2	21 ± 1.3	22 ± 1.2	25 ± 1.2	0.12
MVR (g/ml)	0.7 ± 0.1	0.72 ± 0.16	0.75 ± 0.16	0.87 ± 0.17	0.003
RA Area (cm^2^/m^2^)	12 ± 1.8	12 ± 1.9	11 ± 2.5	11 ± 2	0.69
LA Area (cm^2^/m^2^)^a^	12 ± 1.3	12 ± 1.2	11 ± 1.2	11 ± 1.2	0.38

Bonferroni correction applied- *p* < 0.006 is considered significant

*Abbreviations*: *CKD* chronic kidney disease, *LVEDV* left ventricular end-diastolic volume, *LVESV* left ventricular end-systolic volume, *LVSV* left ventricular stroke volume, *EF* ejection fraction, *LVMht(2.7)* left ventricular mass indexed to height to the power of 2.7, *MVR* left ventricular mass to volume ratio

^a^Logarithmic transformation was applied

**Fig. 1:**
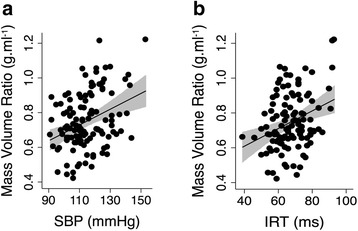
**a** & **b**: Relationship between mass volume ratio (MVR) and cardiovascular characteristics - **a**. MVR versus systolic blood pressure (SBP) (rho = 0.29, *p* = 0.001), **b**. MVR versus isovolumic relaxation time (IRT) (rho = 0.25, *p* = 0.008). The 95% confidence interval of the predicted mean is illustrated by grey zone

The MVR correlated with all measures of BP (SBP: rho = 0.29, *p* = 0.001; MBP: rho = 0.26, *p* = 0.004; and DBP: rho = 0.19, *p* = 0.036; Fig. 1). When a model was created to study the combined effect of BP and CKD group on LV remodeling, both SBP (BP with highest univariate correlation) and CKD group were independent predictors of MVR (*p* = 0.009, *p* = 0.008 respectively).

The SVR correlated with MVR (rho = 0.3, *p* < 0.001), and remained an independent predictor of MVR (*p* < 0.001) in a model including CKD group (*p* = 0.01). There was no correlation between MVR and TAC (*p* = 0.15). Although LVMht^2.7^ did appear higher in CKD, this did not reach statistical significance (*p* = 0.12, Table 4).

There were no differences in EDV, ESV, SV, EF, RAA or LAA between the groups (Table 4).

### Inflow velocities and cardiac timing intervals

The IRT (a marker of active myocardial relaxation [18]) was different across groups (*p* < 0.001, Table 5). On pairwise comparison, IRT was significantly elevated in moderate and severe CKD patients compared to controls (*p* ≤ 0.01). In addition, IRT was higher in severe CKD than in mild CKD patients (*p* = 0.023).

**Table 5:** Cardiac timings and mitral inflow velocities in CKD

	Healthy Controls (*n* = 20)	Mild CKD (*n* = 40)	Moderate CKD (*n* = 40)	Severe CKD (*n* = 20)	*P*-value
IRT (ms)^a^	61 ± 1.2	64 ± 1.1	70 ± 1.1	72 ± 1.1	< 0.001
ICT (ms)^a^	44 ± 1.6	36 ± 1.6	33 ± 1.8	37 ± 1.8	0.24
ET (ms)	277 ± 21	275 ± 16	274 ± 19	276 ± 15	0.92
Tei^a^	0.39 ± 1.3	0.37 ± 1.2	0.38 ± 1.2	0.41 ± 1.3	0.56
E (mls)	47 ± 9.8	49 ± 11	50 ± 11	49 ± 9.7	0.87
A (mls)	19 ± 4.3	20 ± 6.7	21 ± 6.8	22 ± 5.6	0.36
E/A ratio^a^	2.6 ± 1.2	2.6 ± 1.4	2.5 ± 1.5	2.3 ± 1.3	0.63

Bonferroni correction applied- *p* < 0.007 is considered significant

*Abbreviations*: *CKD* chronic kidney disease, *IRT* isovolumic relaxation time, *ICT* isovolumic contraction time, *ET* ejection time, *Tei* myocardial performance index, *E* early transmitral mean velocity, *A* late transmitral mean velocity, *E/A ratio* E to A ratio

^a^Logarithmic transformation was applied

There was a significant association between IRT and MVR (rho = 0.25, *p* = 0.008) as seen in Fig. 1. However, both MVR and CKD group were independent predictors of IRT in a multi-variable model (*p* = 0.01 and 0.007 respectively).

There were no differences in ICT, ET or Tei index between the groups. Peak transmitral E and A wave velocities and peak E/A ratio were also normal in CKD (Table 5).

### Tissue phase mapping

Radial S′ velocity, a marker of systolic function, was significantly different between groups (*p* = 0.003, Table 6). Specifically, radial S′ was lower in moderate and severe CKD compared to controls (*p* ≤ 0.035). There was no significant difference in longitudinal S′ between groups.

**Table 6:** Tissue Phase Mapping in CKD

	Healthy Controls (*n* = 20)	Mild CKD (*n* = 40)	Moderate CKD (*n* = 40)	Severe CKD (*n* = 20)	*P*-value
Rad S′ (cm/s)	2.7 ± 0.31	2.6 ± 0.24	2.5 ± 0.38	2.4 ± 0.31	0.003
Rad E’ (cm/s)	4.2 ± 0.59	3.6 ± 0.62	3.4 ± 0.78	3.3 ± 0.53	< 0.001
Rad A’ (cm/s)	1.3 ± 0.35	1.1 ± 0.34	1.2 ± 0.31	1.2 ± 0.28	0.30
Long S′ (cm/s)	4.6 ± 1.6	3.9 ± 1.1	3.4 ± 1.3	3.6 ± 1.3	0.009
Long E’ (cm/s)	7.9 ± 1.5	7.5 ± 2.0	6.7 ± 1.8	6.8 ± 1.4	0.046
Long A’ (cm/s)	2.3 ± 0.71	2.5 ± 1.1	2.7 ± 0.85	2.5 ± 0.64	0.61
Long E’/A’ ratio^a^	3.4 ± 1.3	3.2 ± 1.6	2.6 ± 1.4	2.8 ± 1.3	0.003^†^
Long E/E’ ratio^a^	6 ± 1.2	6.7 ± 1.3	7.5 ± 1.4	7.3 ± 1.4	0.03

Bonferroni correction applied- *p* < 0.006 is considered significant

*Abbreviations*: *CKD* chronic kidney disease, *Rad* radial, *Long* longitudinal, *S*′ systolic myocardial velocity, *E’* early diastolic myocardial velocity, *A’* late diastolic myocardial velocity, *E’/A’* E’ over A’ ratio, *E/E’* E over E’ ratio

^a^Logarithmic transformation was applied

^†^ANOVA Welch (W) test was used

**Fig. 2:**
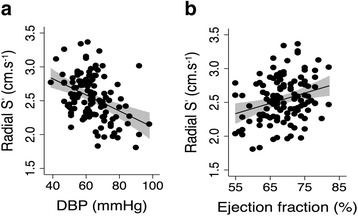
**a** & **b**: Relationship between tissue phase mapping indices and conventional measures of cardiac function - **a**. Radial systolic myocardial velocity (S′) versus diastolic blood pressure (DBP) (rho = − 0.37, *p* < 0.0001), **b**. Radial systolic myocardial velocity (S′) versus ejection function (rho = 0.24, *p* = 0.009). The 95% confidence interval of the predicted mean is illustrated by the grey zone

Radial S′ negatively correlated with MBP (rho = − 0.22, *p* = 0.016), DBP (rho = − 0.37, *p* < 0.0001–Fig. 2) and SVR (rho = − 0.22, *p* = 0.014), but not SBP (*p* = 0.52). Longitudinal S′ did not significantly correlate with any blood pressures. A model created to study the effects of CKD and DBP (BP with highest univariate correlation) on radial systolic velocity demonstrated that DBP was an independent predictor of radial S′ (*p* = 0.001) but CKD group was not significant (*p* = 0.16). Radial S′ also significantly correlated with LVEF (rho = 0.24, *p* = 0.009-Fig. 2), but there was no association between LVEF and longitudinal S′ (*p* = 0.24).

Radial E’ velocity, a marker of LV relaxation and stiffness, was significantly lower in all CKD groups compared to controls (*p* ≤ 0.005) on pairwise comparison. However, there were no significant differences between the CKD groups. There were no significant group differences in longitudinal E’ velocity in CKD (Table 6). Radial E’ was negatively correlated with DBP (rho = − 0.21, *p* = 0.021) and SVR (rho = − 0.27, *p* = 0.003). Two separate models were created to separately study the effects of CKD and DBP or SVR on radial E’. Although CKD group and SVR were independent determinants of Radial E’ (*p* < 0.003 and 0.01, respectively), DBP was not a significant predictor (*p* = 0.19).

In addition, radial and longitudinal E’ correlated negatively with IRT (rho = − 0.28, *p* < 0.003 & rho = − 0.24, *p* < 0.01 respectively). Unlike IRT, neither was associated with MVR or LVMht^2.7^ (*p* > 0.25).

The E’/A’ was different between groups (*p* = 0.003, Table 6), but was only significantly reduced in moderate CKD compared to controls and mild CKD (*p* ≤ 0.035) on pairwise comparison. There were no group differences in radial and longitudinal A’ velocities or E/E’.

### Association between renal and cardiovascular biomarkers

Worsening eGFR was associated with higher MBP (rho = − 0.26, *p* = 0.04), MVR (rho = − 0.29, *p* = 0.02) and longer IRT (rho = − 0.30, *p* = 0.02). On multi-variable regression, eGFR remained a significant determinant of MVR (β = − 0.26, *p* = 0.035,) and IRT (β = − 0.28, *p* = 0.043) after adjustment for MBP (*p* ≥ 0.06). In a model with IRT as dependant variable, eGFR trended towards significant (β = − 0.25, *p* = 0.052) and MVR was significant (β = 0.27, *p* = 0.039).

Reduced Hb was also associated with a reduced radial function (Rad S′: rho = 0.35, *p* < 0.005, Rad E’: rho = 0.35, *p* < 0.006). These relationships remained significant after adjusting for the effect of afterload (MBP or SVR) in a multi-variable regression model (Rad S′ models: Hb *p* = 0.001, Rad E’ models: Hb *p* = 0.001). There were no other significant relationships between eGFR, PTH and Hb with other cardiovascular parameters.

## Discussion

This is the largest study to investigate the vascular and cardiac phenotype in pre-dialysis pediatric CKD using CMR. The main findings were that children with CKD had: i) elevated BP (both MBP and DBP), ii) increased SVR, but no evidence of increased vascular stiffness, iii) higher LV MVR independent of BP, iv) abnormalities of diastolic function suggestive of reduced active relaxation and increased chamber stiffness and v) Reduced systolic velocities with preserved global systolic function. These changes appeared most marked in children with moderate and severe CKD stages.

### Vascular phenotype in pediatric CKD

It is well recognized that hypertension is common in pediatric CKD [19]. However, the abnormal components of afterload have not been fully described. In this study, we found that SVR was significantly increased in all CKD groups compared to normal controls. Possible reasons include; salt retention and abnormal renin-angiotensin stimulation [20], sympathetic overdrive [21] and endothelial dysfunction via reduced nitric oxide bioavailability [22]. Irrespective of the cause, our results suggest that vascular resistance is an important mediator of increased afterload and may be a useful target for intervention. Interestingly, we found no evidence of reduced TAC or increased aortic stiffness. This is slightly surprising as studies have shown elevated pulse wave velocity (PWV), reduced carotid distensibility and increased carotid intimal medial thickness in children with CKD [23]. However, elevated PWV is not a universal finding and even in positive studies, PWV is only mildly raised (i.e. in the 4C’s study the PWV z-score was 0.33) [23]. Furthermore, changes in carotid artery characteristics do not necessarily reflect changes in the aorta [24], which is the repository for most vascular buffering. In fact, pulse pressure has been shown to be normal in other large pediatric CKD studies [25], which is in keeping with our finding of normal aortic stiffness. Of course, the vascular phenotype may be different in dialysis patients due to more extensive arterial calcification.

Other possible causes of hypertension in CKD are fluid overload or high CO. Although fluid overload is difficult to assess, we did show that RAA (a marker of fluid overload) was normal in our cohort. Furthermore, we also demonstrated that CO was not increased in CKD. Therefore, it is unlikely that these factors are important in the pathogenesis of hypertension in early CKD.

### Left ventricular remodelling in CKD

In our study, we demonstrated that children in the severest CKD group had increased LV MVR compared to controls and, as eGFR fell, MVR increased. These results suggest that worsening CKD is associated with increased concentric remodelling in children. Interestingly, height indexed LV mass was not statistically different in our population, unlike previous TTE studies. This may be due to the well-recognized overestimation of LV mass using TTE, particularly in patients with CKD [26].

Ventricular remodelling in these children is partly explained by their hypertensive phenotype. However, both CKD group and eGFR remained significant predictors of MVR after controlling for BP. This implies that other ‘uremic’ processes are involved in CKD associated remodelling. Possible ‘uremic’ causes of hypertrophy include: anemia, hyperparathyroidism and sympathetic overactivity [24, 27, 28]. Identification of the exact stimulus may provide new targets for therapeutic intervention that may be important in remediating the increased cardiovascular risk associated with LVH [2].

### Diastolic function in CKD

We found conventional transmitral inflow peak E/A to be normal in children with CKD. However, both IRT and radial E’ were abnormal, pointing towards subtle diastolic dysfunction in moderate to severe CKD. In addition, longer IRT was associated with lower eGFR. IRT is a marker of active myocardial relaxation and correlates with invasively measured isovolumic relaxation constant [18]. Previous studies in adults have shown that active relaxation is associated with LV mass [29] and our findings are in keeping with this. However, even after controlling for MVR, CKD remains a significant predictor of IRT and eGFR trends towards significance. This suggests that CKD exerts an independent effect possibly mediated through abnormal energy or calcium handling. This could be due to myocyte/capillary mismatch resulting in a relative oxygen deficit as demonstrated in animal models [30].

Early diastolic peak velocity is a marker of both active relaxation and ventricular compliance [31]. Our demonstration of lower E’, measured using TPM, is in keeping with a previous smaller study in pediatric CKD [32]. In our study, E’ did not correlate with MVR or LVMht^2.7^. Thus, reduced early filling cannot simply be explained by LV hypertrophy. One explanation for reduced ventricular compliance is myocardial fibrosis, which has been demonstrated in several animal models [33]. Unfortunately, it is not possible to perform post contrast T1 mapping in these patients due to the risk of nephrogenic systemic fibrosis [34]. Nevertheless, non-contrast methods are now available [34] and these could be used to quantify fibrosis in future studies. We also demonstrated that lower radial E’ was associated with lower Hb. The reason for this is unclear but may be related to reduced oxygen delivery affecting the active component of relaxation.

### Systolic function in CKD

Global systolic function was preserved in CKD patients with normal LVEF. However, radial S′ was reduced in moderate and severe CKD, implying some element of systolic dysfunction in the latter stages of renal disease. These findings are in keeping with previous tissue Doppler imaging and echocardiographic strain studies and illustrate the importance of assessing systolic function in a more sophisticated manner [4, 35]. The most obvious explanation for reduced S′ is that increased blood pressure limits contraction through the force velocity relationship. This is in keeping with our findings in which DBP independently predicted radial S′, unlike CKD group.

### The use of CMR for cardiovascular assessment in CKD

We have demonstrated that children with CKD exhibit a specific, but subtle cardiovascular phenotype. This phenotype is characterized by: i) increased MVR, ii) increased IRT, iv) reduced radial S′ and E’ and iv) increased SVR. All of these metrics can be evaluated with TTE. In fact, due to its high temporal resolution, it is probably the most accurate method of assessing timing parameters (i.e. IRT) and tissue velocity. However, there are some limitations to TTE. In particular, TTE overestimates LVM [26] and cannot accurately estimate CO (necessary for calculation of SVR). As we have found that these two measures are abnormal in pediatric CKD, we believe there may be a role for CMR identification of abnormal cardiac and vascular phenotypes in this group of patients. Nevertheless, it is still unclear what the clinical significance of this phenotype is. The next important step is to ascertain whether it can be used to guide therapy and determine prognosis.

### Limitations

An important limitation is the relatively older age of the study cohort. Although the entire cohort of CKD patients did include patients from 7 to 18 years of age, the majority of children were between the ages of 9 to 16 years. Thus, the study findings may not be as applicable to younger children. In order to ensure that the study findings can be extrapolated to all age groups, future studies should consider recruiting younger patients.

The absence of recent TTE data in the subjects is also a potential limitation in this study. Performing an TTE in tandem may have been informative for the comparison of CMR findings with an established standard clinical investigation. This would have enabled us to confirm our CMR findings of subtle systolic and diastolic impairment with TTE results. Unfortunately, this was not performed as part of the study protocol in order to minimise inconvenience to the study subjects. No recent clinical TTE data were available for comparison in this cohort either. However, despite that, our findings are in agreement with the majority of previous studies in pediatric CKD using TTE as referenced in the paper.

Another limitation is our use of mild, moderate and severe CKD grouping instead of the conventional categorization of CKD stages 1–5. This was done to ensure adequate powering for group wise differences. However, the use of conventional CKD groups would be desirable in future larger, multicenter studies.

Finally, BP measurements were carried out using an oscillometric sphygmomanometer, which may be less accurate than using manual sphygmomanometer [36]. This was because it was essential for BP measurements to be acquired simultaneously during PCMR flow acquisition for accurate assessment of vascular measures. Unfortunately, this could not be performed manually in the CMR scanner during flows acquisitions. However, BP measurement was taken with a standardized protocol for all subjects using the same machine. This meant that the measurements were comparable across the cohort.

## Conclusion

It is possible to use CMR to comprehensively evaluate cardiac and vascular phenotype in children with CKD. In the future, these novel CMR indices may even be useful for identifying high-risk pediatric CKD patients. More work is needed to determine normative pediatric values. Future prospective studies will also be required to correlate these markers with prognosis in order to predict cardiovascular risk.

## Additional file

Additional file 1: This table outlines the clinically relevant blood and urine tests in the mild, moderate and severe CKD groups. (DOCX 17 kb)

## Abbreviations

A: Peak late diastolic (mitral inflow) velocity
A’: Peak late diastolic LV myocardial velocity
ANOVA: Analysis of variance
AoC: Ascending aortic compliance
AoMax: Maximum ascending aortic diameter
AoMin: Minimum ascending aortic diameter
AoS: Ascending aortic strain
BP: Blood pressure
BSA: Body surface area
CKD: Chronic kidney disease
CMR: Cardiovascular magnetic resonance
CO: Cardiac output
DBP: Diastolic blood pressure
E: Peak early diastolic (mitral inflow) velocity
E’: Peak early diastolic LV myocardial velocity
EDV: End-diastolic volume
EF: Ejection Fraction
eGFR: Estimated glomerular filtration rate
ESV: End-systolic volume
ET: Ejection time
FOV: Field of view
Hb: Hemoglobin
ICT: Isovolumic contraction time
IRT: Isovolumic relaxation time
LV: Left ventricle/left ventricular
LVH: Left ventricular hypertrophy
LVM: Left ventricular mass
LVMht^2.7^: Left ventricular mass indexed to height to the power of 2.7
MBP: Mean blood pressure
MVR: Mass volume ratio
PCMR: Phase contrast cardiovascular magnetic resonance
PP: Pulse pressure
PTH: Parathyroid hormone
PWV: Pulse wave velocity
Rho: Spearman’s coefficient
S′: Peak systolic LV myocardial velocity
SBP: Systolic blood pressure
SV: Stroke volume
SVR: Systemic vascular resistance
TAC: Total arterial compliance
TE: Echo time
TEI: Myocardial performance index
TPM: Tissue phase mapping
TR: Repetition time
TTE: Transthoracic echocardiography
VENC: Maximum measurable velocity range
β: Beta coefficient of variables in regression model
