# Supplementary material for: A comprehensive characterization of myocardial and vascular phenotype in pediatric chronic kidney disease using cardiovascular magnetic resonance imaging
Source: J Cardiovasc Magn Reson. 2018 Mar 29;20:24. doi: 10.1186/s12968-018-0444-0 (PMC5880006; doi:10.1186/s12968-018-0444-0)
Supplement: Supplementary file 1 — This table outlines the clinically relevant blood and urine tests in the mild, moderate and severe CKD groups. (DOCX 17 kb) [file 12968_2018_444_MOESM1_ESM.docx]

**Additional file 1**

Table S1: Clinical blood and urine test results of study population.

|  | Mild  CKD  (n=40) | Moderate CKD  (n=40) | Severe CKD  (n=20) | P-value |
| --- | --- | --- | --- | --- |
| **Bloods tests** |  |  |  |  |
| Hemoglobin (g/L) | 132±13 | 133±16 | 128±11 | 0.57 |
| Hematocrit (L/L) | 0.38±0.04 | 0.38±0.04 | 0.37±0.03 | 0.61 |
| Platelet count (x10^9^/L)* | 295±1.3 | 247±1.3 | 224±1.3 | 0.006 |
| White Cell Count (x10^9^/L) | 6.9±2.4 | 6.5±1.6 | 7.0±2.5 | 0.72 |
| Sodium (mmol/L) | 143±2.4 | 143±2.7 | 143±3 | 0.79 |
| Potassium (mmol/L)* | 4.1±1.1 | 4.2±1.1 | 4.3±1.1 | 0.51 |
| Urea (mmol/L) | 5.4±1.2 | 8.6±2.5 | 18.0±5.2 | <0.001† |
| Creatinine (umol/L)* | 68±1.2 | 117±1.2 | 305±1.4 | <0.001† |
| eGFR (ml/min/1.73 m^2^) | 79±8.8 | 48±8.4 | 19±6.5 | <0.001 |
| Calcium (mmol/L) | 2.4±0.095 | 2.4±0.11 | 2.4±0.16 | 0.999 |
| Phosphate (mmol/L) | 1.4±0.18 | 1.4±0.16 | 1.5±0.28 | 0.38 |
| Calcium-Phosphate product (mmol^2^/L^2^) | 3.4±0.45 | 3.4±0.39 | 3.5±0.56 | 0.40 |
| Ionised Calcium pH corrected (mmol/L) | 1.2±0.054 | 1.2±0.065 | 1.2±0.054 | 0.92 |
| Intact parathyroid hormone (pmol/L)* | 3.5±3.2 | 3.7±2.4 | 8.5±2.4 | 0.011 |
| **Urine Tests** |  |  |  |  |
| Urine Albumin (mg/L)* | 26±5.6 | 119± 6.4 | 747±3.2 | <0.001 |
| Urine Creatinine (mmol/L)* | 5.4±2.3 | 5.4±2.3 | 2.7±1.7 | 0.13 |
| Urine Albumin/ Creatinine Ratio (mg/mmol)* | 4.7±3.7 | 22±6.2 | 261±2.5 | <0.001 |

^*- Logarithmic transformation was applied. †- ANOVA Welch (W) test was used. Abbreviations: CKD= chronic kidney disease. Only 65 and 46 CKD patients had blood and urine tests respectively within 3 months of the study, while 48 and 58 patients had recent ionized calcium and parathyroid hormone levels respectively.^
